# Physician assisted-dying in mentally and somatically ill individuals in Switzerland: Protocol for survey-based study

**DOI:** 10.3389/fpsyt.2022.987791

**Published:** 2022-10-28

**Authors:** Eva Kowalinski, Tiziana Ziltener, Sarah Staub, Julian Moeller, Manuel Trachsel, Andres R. Schneeberger, Irina Franke, Undine E. Lang, Christian G. Huber

**Affiliations:** ^1^Department of Adult Psychiatry, University Psychiatric Clinics (UPK), University of Basel, Basel, Switzerland; ^2^Division of Clinical Psychology and Epidemiology, Department of Psychology, University of Basel, Basel, Switzerland; ^3^Clinical Ethics Unit, University Hospital Basel (USB), Basel, Switzerland; ^4^Biomedical Ethics, Institute of Biomedical Ethics and History of Medicine, University of Zurich, Zurich, Switzerland; ^5^Behavioral and Mental Health Care, University of San Diego, San Diego, CA, United States; ^6^Department of Psychiatry and Behavioral Sciences, Albert Einstein College of Medicine, Bronx, NY, United States; ^7^Department of Adult Psychiatry, Psychiatric Hospital, University of Zurich, Zurich, Switzerland; ^8^Departments of Adult and Forensic Psychiatry, Psychiatric Services Grisons (PDGR), Chur, Switzerland; ^9^Department of Forensic Psychiatry and Psychotherapy, Ulm University, Ulm, Germany

**Keywords:** severe mental disorder, medical ethics, terminal illness, intolerable suffering, decision-making capacity, stigmatization, assisted suicide

## Abstract

**Background:**

Article 115 of the Swiss Penal Code (StGB) permits physician-assisted dying (PAD), provided it is not performed for “selfish reasons,” and thus, occupies a special role in international comparison. However, the Swiss federal law does not regulate who exactly is entitled to access PAD, and there is no universal agreement in the concerned professional societies. Additional uncertainty arises when assessing the wish for PAD of a mentally ill person compared to a somatically ill person.

**Objectives:**

This study aims to contribute to the discussion of PAD among the mentally ill and to provide insight into the current situation in Switzerland.

**Methods:**

This is a monocentric prospective observational survey-based study. We will conduct an exploratory online/telephone survey about PAD in somatic vs. mental illness in Switzerland. The survey sample will comprise 10,000 Swiss residents of the general population from all three language regions (German, Italian, and French) as well as 10,000 medical professionals working in the seven states (“cantons”) of Basel-Stadt, Basel-Landschaft, Aargau, Lucerne, Graubünden, Ticino, and Vaud. Opinions on PAD in mentally and somatically ill patients will be assessed using 48 different case vignettes. Each participant will be randomly assigned a somatic terminal, a somatic non-terminal, and a mental non-terminal case vignette. Furthermore, the attitude toward the ethical guidelines of the Swiss Medical Association of 2004, 2018, and 2022, as well as the stigmatization of mentally ill people will be assessed.

**Discussion:**

Physician-assisted dying in mentally ill persons is a highly relevant yet controversial topic. On the one hand, mentally ill persons must not be discriminated against in their desire for PAD compared to somatically ill persons while at the same time, their vulnerability must be considered. On the other hand, treating physicians must be protected in their ethical integrity and need security when judging PAD requests. Despite its relevance, data on PAD in the mentally ill is sparse. To regulate PAD for the mentally ill, it is therefore important for Switzerland—but also internationally—to gain more insight into the ongoing debate.

**Clinical trial registration:**

ClinicalTrials.gov, identifier: NCT 05492461.

## Introduction

While active-direct euthanasia (killing on demand) is prohibited in Switzerland under Art. 114 of the Swiss Penal Code (StGB), physician-assisted dying (PAD) is not regulated by the law (Art. 114 section 1 StGB). Art. 115 of the StGB prohibits assisted dying only if it is performed for “selfish motives” (Art. 115 section 1 StGB). In our study, we use the term PAD for the concept of physician-assisted suicide as it is performed in Switzerland.

The Swiss Academy of Medical Sciences (SAMS) does not consider PAD as a medical activity but respects it as a “legally permissible activity” that a physician may perform as a “personally responsible decision” after careful examination of the necessary prerequisites. The SAMS (1) lists the following necessary criteria for the granting of PAD: a patient must have preserved decision-making capacity (DMC) regarding the wish to die. “It must be documented that incapacity has been carefully excluded by the physician.” The wish to die must be self-determined, deliberate, and permanent. If a patient is dependent on people in his environment, their possible influence on the wish to die must be carefully considered. Furthermore, the comprehensibility or justifiability of the wish to die in the patient's concrete individual life situation must be examined. Medically indicated treatment options, as well as alternative support options, must have been sought and exhausted or must have been declared unacceptable by the patient who was capable of decision-making in this regard. In addition, a patient's suffering must be classified as “intolerable” according to the current version of the SAMS ethical guidelines (2). The term intolerable suffering replaced the term terminal illness, which was valid as a prerequisite for PAD until 2018 (3). However, intolerable suffering is still not a sufficient criterion for PAD; preserved DMC and exhausted or unacceptable alternative options are additional necessary prerequisites (4).

A patient's wish for PAD can lead to the professionals perceiving a moral conflict between the ethical principle of respect for patient autonomy, and the ethical principle of beneficence in the sense of preserving the patient's life (ethical principles according to Beauchamp and Childress) (5).

When assessing the legal requirements for PAD, additional difficulties arise when a patient is suffering from a mental disorder compared to a somatic illness. Due to the mental disorder, DMC and resistance to possible external pressure influencing the wish to die may be impaired. This might raise doubts about the appropriateness and self-determination of the wish to die (6). Furthermore, most mental disorders cannot be classified as terminal illnesses. In many situations, the assessing psychiatrist might see alternatives other than death, such as the possibility of recovery or numerous treatment options (especially if he or she tends to view suicidality as a potentially treatable symptom of the illness). This complicates an unbiased assessment of the patient's judgment (7).

In the last years, PAD practices have expanded internationally. While in Switzerland and the Benelux countries PAD practices have been legal for a long time, other European countries such as Germany and Austria have recently changed their jurisdiction while the terms and conditions of implementation are not available yet (8, 9). Outside of Europe, PAD is legal in Colombia, Canada, three states in Australia, and 11 states in the USA. Nevertheless, assisted dying is subject to controversial medical ethics and public debate internationally, and also in Switzerland. For example, the law does not specify who may get access to PAD, and professional societies disagreed on this subject for 3 years: while intolerable suffering was accepted as a key criterion by the SAMS, the Swiss Medical Association (FMH) rejected this because it deemed the term “intolerable suffering” as imprecisely defined, insufficiently delimitable, and too strongly dependent on the subjective assessment of the patient. Therefore, the decision on granting assisted dying may hold great uncertainty for the physicians in charge. Consequently, the FMH postulated the following: “Physician-assisted dying should therefore be limited to those patients who are suffering from a fatal illness and whose condition will not improve with proper medical treatment. Such a diagnosis can be made by a physician with sufficient reliability” (10). This requirement particularly discriminated against mentally ill patients with a prolonged wish to die, since although they can suffer intolerably in the same way as somatically ill patients, their illness is not typically classified as terminal (10, 11). In May 2022, as a result of the cooperation of the SAMS and the FMH, both institutes accepted an updated version of the guidelines. As in the former version of 2018 they require that the intolerable suffering is caused by symptoms of a disease and as a new complement the severity of the condition must be substantiated by an appropriate diagnosis and prognosis (2).

The Benelux countries and Canada contradict the FMH in that they do not tie PAD to end-of-life and accept suffering from mental illness as a justification for assisted dying (6). The FMH also considers suicide prevention to be jeopardized by the extension of assisted dying to non-terminal patients, which is particularly important in the case of mentally ill patients, in whom the wish to die may be an expression of a treatable mental disorder and may not be a self-determined, well-considered, and permanent choice by a person (10, 11). It is therefore of great importance to determine the patient's DMC. The assessment of DMC must consist of a systematic evaluation and careful justification. Only in this way, the dilemma of respecting patient autonomy and protecting vulnerable persons can be resolved (12, 13). Mental disorders may sometimes, but not always be accompanied by a lack of DMC. Moreover, impaired DMC may be limited to acute episodes of the disorder and the patient may be capable of making decisions during symptom-free intervals. Furthermore, DMC may not be affected globally and can be limited to specific areas, which is referred to as the “situational relativity” of DMC (14).

It is up for debate whether the stigmatization faced by individuals with mental illness influences a physician's evaluation of their DMC. Often, individuals with mental illness are seen as dangerous, unpredictable, and violent, and their self-reports are doubted (15). In addition to the general stigmatization, the values, prejudices, and attitudes of the evaluating physician can also influence the assessment of PAD (16, 17). Although non-terminal, mental illness can cause equally severe suffering. It would be inconsistent and discriminatory against persons with mental illness if physical and mental suffering were not regarded as equivalent and individuals with mental illness were excluded *per se* from assisted dying under the guise of suicide prevention. Therefore, it is up for debate whether only a terminal state of illness is an appropriate justification criterion for PAD or whether the poor quality of life and the presence of suffering cannot be comparably crucial criteria (14). However, not only the quality and quantity of the suffering but also the patient's entire life situation and the motives behind his or her wish to die should be included in the consideration (18). In this context, the motives behind the wish for assisted dying of psychologically and somatically ill patients may not differ substantially, and both include psychological suffering (19).

Since 2008, the number of registered cases of PAD in Switzerland increased steadily. In 2014, 724 cases of assisted dying were recorded. This is 26% more than the year before and 1.2% of all deaths. In about half of the cases, cancer was the cause, and in about 3%, a mental illness (20).

It is essential to keep in mind, however, that in cases where somatic illness is cited as the main reason for the wish for assisted dying, it is not uncommon to find psychological comorbidity. This psychological suffering can contribute significantly to the wish to die. The proportion of mental illnesses is therefore likely to be underestimated (21).

Thus, while a somatic illness is most often the official causing factor for the wish to die, data from the Netherlands indicate an increase in PAD among those with mental illness (6).

### Importance of the research question

The regulation of PAD in psychiatric patients is subject to controversial discussions. It presents an extraordinarily challenging situation in medical practice and is of increasing relevance both for Switzerland and internationally, as evidenced by the rising incidence of PAD in mentally ill patients (see above). However, there is currently little scientific data on this issue (6).

With the present study, we want to contribute to the discussion regarding the need of regulating PAD in mentally ill patients. On the one hand, mentally ill patients with a wish to die must not experience any disadvantage compared to somatically ill patients, whilst at the same time, their vulnerability must be considered. On the other hand, treating physicians must be protected in their ethical integrity. Using an anonymous online survey, we aim to assess the attitude of the general population and medical professionals toward the current medico-ethical guidelines of the SAMS. Furthermore, we want to examine to what extent the public discussion corresponds to the current situation in clinical practice. The results of the study may also indicate to what extent stigmatization needs to be counteracted through public education or continuing medical education, should the survey show that prejudices against mentally ill patients are linked to the assessment of their wish to die.

### Primary objective

The current study investigates the acceptance of PAD in the general population on the one hand, and in the medical profession, on the other hand, depending on (1) the type of illness (somatic/mental), (2) the presence of intolerable suffering, (3) DMC, (4) the availability of therapeutic options, and (5) the terminal nature of the illness based on case vignettes. Furthermore, it examines to what extent the stigmatization of mentally ill persons is linked to the acceptance of their wish to die. It is an exploratory analysis to provide first insights into the current situation in Switzerland, paving the way for further research projects to form hypotheses. Within the scope of this project, the following research questions will be investigated:

1. Are there associations between the type of illness (somatic vs. mental) and the decision on whether a person should be granted access to PAD?
2. Does the reasoning behind accepting the legitimacy of wishes to die correspond to the conflict between professional societies (terminal illness vs. intolerable suffering)?
3. To what extent does the acceptance of PAD in concrete situations described in case vignettes correspond to the positioning on the SAMS criteria of 2004, 2018, and 2021?
4. Which criteria do participants from the public and the medical profession indicate as relevant to them in an open answer format?
5. Is there an association between the level of stigmatization and the assessment of whether a person should be granted access to PAD?
6. Are there differences between medical professionals and the public regarding research questions 1–5?

## Methods

### Study design

This is a monocentric prospective observational survey-based study. The study is exploratory and will consist of an online/telephone survey about PAD in somatic vs. mental illness in Switzerland on a sample of the general population from all three language regions (German, Italian, and French) as well as medical professionals working in the seven states (“cantons”) of Basel-Stadt, Basel-Landschaft, Aargau, Lucerne, Graubünden, Ticino, and Vaud. Each participant will be randomly assigned a somatic terminal, a somatic non-terminal, and a mental non-terminal case vignette. Furthermore, the attitude toward the ethical guidelines of the Swiss Medical Association of 2004, 2018, and 2022, as well as the stigmatization of mentally ill people will be assessed. The study questionnaire is described in detail below.

### Study questionnaire

#### Demographic information

The following demographics will be collected in both groups at the beginning of the questionnaire:

- gender and age
- household composition and marital status
- nationality
- the highest level of education and current occupation
- religious beliefs
- experience with psychiatric treatment in their closer environment (family, friends)
- experience with PAD/suicide in the immediate environment (family, friends)
- familiarity with Swiss legislation on PAD.

In addition, physicians will be asked about their professional specialty and experience with PAD/suicide in their professional context. Apart from these two questions, the questionnaires are identical for the two groups. At the beginning of the survey, participants from the general population will be asked if they work as a physician. If the question is affirmed, participants will only be requested to complete the questionnaire if they have not done so yet as part of the medical professional's group. If a physician from the general population has not yet filled in the questionnaire, they will be asked to participate, and the data set will then be assigned to the group from the medical profession for analysis.

#### Case vignettes and assessment of PAD

It is essential that the persons taking part in the study fully understand what is meant by PAD and this cannot be taken for granted when addressing the general population. Physician-assisted dying is therefore explained in lay terms before the questions concerning PAD are posed.

For the vignettes, three cases were created: one about a person suffering from a non-terminal somatic illness, one about a person suffering from a terminal somatic illness, and one about a person suffering from a non-terminal mental illness. For each of these cases, there will be 16 different versions. Common to all of them will be the three factors of well-consideration, self-determination, and permanence of the desire to die. In contrast, the factors tolerability vs. intolerability of suffering, DMC vs. incapacity, the absence vs. presence of a therapeutic option, and the gender of the person depicted differ and are permuted with each other in the vignettes. This will result in a total of 48 different case vignettes. Expert ratings will be used to assess the interrater reliability of the case vignettes. To ensure that the assessment of different raters has a concurrent outcome, interrater reliability is calculated using Fleiss' kappa with a target value of at least 0.8, indicating substantial agreement (22). This measure has the advantage over other kappas such as Cohen's kappa that interrater reliability by more than two raters can be tested.

Each participant will be randomly assigned three case vignettes—one (out of 16 possible variations) about a person suffering from a non-terminal somatic illness, one (out of 16 possible variations) about a person suffering from a terminal somatic illness, and one (out of 16 possible variations) about a person suffering from a non-terminal mental illness. For these three concrete situations, the participant will be asked to assess whether physician-assisted suicide should be granted or not. The participant will define the degree of his or her approval or disapproval on a 5-point Likert scale and will be asked to justify the decision by describing his or her motives in an open answer format. Furthermore, participants will be asked to select those items from a list of alternative justifications that are decisive for their judgment.

Using the online tool Random.org (https://www.random.org/sequences/), case vignettes will be randomized and assigned to participants via a randomly generated sequence based on the address lists.

Furthermore, we aim to assess participants' opinions on the SAMS ethical guidelines of 2004, 2018, and 2021. For this purpose, participants will be presented with the guidelines and will be asked to indicate their degree of agreement on the criteria using a 5-point Likert scale (1 = complete rejection, 5 = complete agreement).

#### Survey instruments

The stigmatization of persons with a mental illness will be assessed using, on one hand, the Bogardus scale (23). This test scale measures, as an indicator of stigmatization, the participant's desire for social distance toward the mentally ill. It asks about the willingness to engage in social interaction with a person who suffers from a mental disorder for the following seven situations of increasing closeness: subtenancy, co-worker, neighbor, babysitter of own child, spouse of a family member, and member of own social group. Participants indicate their level of agreement with a score between 1 and 4, a lower score indicating lower acceptance. The German translation of the Bogardus scale has already been used successfully in studies (24). On the other hand, the acceptance of coercive measures in psychiatry will be assessed to estimate the stigmatization of mentally ill patients. For this purpose, the Staff Attitude to Coercion Scale (SACS) will be used. A recently published study adapted the scale for the German language and could show that this version provides valid results to assess the influence of staff attitudes toward coercive measures in psychiatry on their actual use (25).

### Sample characteristics

For this study, we will survey a group of 10,000 Swiss residents from the general population including the three language regions (German, Italian, and French) as well as a group of 10,000 physicians practicing in the states (“cantons”) of Basel-Stadt, Basel-Landschaft, Aargau, Lucerne, Graubünden, Ticino, and Vaud. In total, the sample thus comprises 20,000 individuals. For each of the two groups, we performed a case number estimation to determine the size of a significant sample. For the group from the general population, our case number estimation showed that a sample size of *n* = 2,373 yields significant results at a 95% confidence interval and a margin of error of 2%. Based on the literature and our empirical values, we assume a response rate of 30%, which corresponds to 3,000 valid records for 10,000 persons and thus harmonizes with our estimated representative sample size. We also performed a case number estimation for the group of medical professionals, which indicated a sample size of *n* = 2,258 as significant at a 95% confidence interval and a margin of error of 2%. According to the 2019 FMH statistics, we plan to survey 10,000 physicians. Therefore, with a response rate of 30%, we can expect 3,000 valid data sets.

Eight thousand residents from the state of Basel-Stadt are to be randomly drawn from the state's register and contacted via postal mail. Furthermore, a sample of 2,000 individuals of the general population from the Italian- and French-speaking parts of Switzerland will be recruited by a polling institute. For the survey of the 10,000 physicians from Basel-Stadt, Basel-Landschaft, Aargau, Lucerne, Graubünden, Ticino, and Vaud we rely on the cooperation with the responsible health departments.

### Eligibility criteria

The inclusion criteria for participants from the general population are (1) Swiss residents between 18 and 65 years old, and (2) sufficient knowledge of German, Italian, or French. Medical professionals are included if they are registered in one of the states (“cantons”) Basel-Stadt, Basel-Landschaft, Aargau, Lucerne, Graubünden, Ticino, or Vaud. Physicians from all specialties are to be surveyed to be able to draw a comparison between the different medical professionals.

### Data collection methods

The online survey will be created and administered using the online survey tool “Unipark” (Tivian XI GmbH, released 2020, Unipark, Köln, Germany: Tivian XI GmbH). The survey data will then be exported to Excel for storage.

The telephone survey in the Italian- and French-speaking parts of Switzerland will be conducted by a polling institute. Telephone survey data will be entered into the Unipark online survey as well and extracted to Excel for storage.

Project data will be handled with the uttermost discretion and is only accessible to authorized personnel who require the data to fulfil their duties within the scope of the research project. On the Case Report Forms (CRFs) and other project-specific documents, participants are only identified by a unique participant number. Data will be stored and used according to the Swiss Data Protection Act. The survey is anonymous, and no identifiable information will be stored. The data will be stored in an encrypted and password-protected Excel file which only the research team will have access to. They are subject to the duty of confidentiality. The data is stored in the internal clinical data system which aligns with the security standards for patient data in Switzerland.

In addition, we will regularly save a copy of this file on a CD to be able to track changes in the data at a later point in time. We keep dated and signed CDs in a safe. Only the research team can open this safe. To allow follow-up studies, the anonymized data will be kept indefinitely.

### Survey administration

Participants from the general population will be recruited via postal mail as well as via a polling institute. Medical professionals will be recruited and contacted via email.

Those participants from the general population who will be contacted by postal mail will receive a QR code that will take them to the online survey. Participants from the medical profession will be directed to the online survey via a link that they will receive by email. The QR code or link will be used to access the survey start page, where the study will be described in detail and individuals are informed regarding participation. To start study participation, individuals must click the consent button. Only then will the survey begin. Individuals who do not wish to participate may, but are not required to, provide a reason. Persons from the general population contacted by telephone will be verbally informed about the study and asked for their consent before the survey begins.

Participants can complete the online questionnaire within 3 months. Once the survey period will be completed, the data will be analyzed within approximately 3 months, discussed, and summarized in a publication within another 3 months. In total, the research project is planned to take 14 months.

### Statistical analysis

The primary endpoint will be the degree of consent to PAD depending on the type of illness (somatic vs. mental).

In addition, the following secondary endpoints have been defined:

1. Degree of consent to PAD as a function of
a. the intolerable suffering
b. DMC
c. the availability of therapeutic options
d. the terminal nature of the illness.
2. Positioning of the respondents to the SAMS criteria of 2004, 2018, and 2021.
a. Spontaneously expressed opinions in an open answer format in the context of a qualitative evaluation.
b. Agreement or disagreement when presented SAMS criteria of 2004, 2018, and 2021.
3. Association between the positioning of the respondents on the SAMS criteria of 2004, 2018, and 2021, and the evaluation of the case vignettes.
4. Association between the degree of stigmatization and the assessment of PAD.
5. Comparison of the primary and secondary endpoints between the two groups.

The statistical analysis of the collected data will be done with a standard statistical package, either IBM SPSS Statistics (IBM Corp, released 2020, IBM SPSS Statistics for Windows, Version 27.0, Armonk, NY: IBM Corp.) or R Studio (RStudio Team, released 2020, RStudio: Integrated Development for R. Boston, MA: RStudio, PBC). The descriptive statistics of the sample characteristics will be performed separately for the two groups and the whole sample. For binary and categorical variables, we will report frequencies in numbers and percentages; for continuous variables, we will report the mean and standard deviation, and the median and interquartile range, respectively. Given the fact that our research project is an exploratory study, we do not explicitly specify significance levels. The primary endpoint [level of PAD consent as a function of the type of illness (somatic vs. mental)] will be evaluated in a simple regression analysis with the level of PAD consent as the dependent variable and type of illness as the independent variable. In a multiple regression analysis, we will further examine the association of demographic factors such as gender with the dependent variable. The variables general population/medical professional and gender will be controlled for statistically whenever necessary. The secondary endpoints will be evaluated—as far as possible—with the same analyses. To explore the responses from the free-text fields, computer-assisted content analysis will be used (MAXQDA12; VERBI Software GmbH, Berlin, Germany).

We will test for differences between the two groups using two-tailed *t*-tests (in the presence of a normal distribution) or the Mann-Whitney U test (in the absence of a normal distribution) for continuous variables and the Chi^2^-test for categorical variables.

The model assumption will be checked in advance and the distributions of the endpoints will be plotted with scatter plots. If any model assumptions are not met, confidence intervals will be created using bootstrap. This resampling-based, non-parametric procedure can also be used if the model's normal distribution assumptions are not met.

If a participant completes the online survey entirely, missing values are not to be expected, as the survey will be designed in a manner such that it can only be completed if all fields are filled in. Missing values in incomplete surveys can be considered “data missing at random” and estimated via multiple imputations. Outliers will be identified and checked for their plausibility. If a participant drops out of the online survey this will be registered and the dropout rates of both groups will be explored. By choosing an appropriately large sample size response dropouts have been accounted for.

## Ethics statement

The current study has been approved by the ethics committee in charge [Ethics Committee Northwest and Central Switzerland (EKNZ)] and conforms to all relevant ethical regulations (Ethics Committee Number: Req-2021-01396). Participation in the survey is voluntary after full disclosure of the measures regarding data protection. Before the start of the survey, all participants were informed about their study and gave their informed consent.

## Author contributions

EK, TZ, and CH designed the study. EK, TZ, and SS wrote the initial draft of the paper. JM, MT, AS, IF, and UL critically revised the manuscript for important intellectual content. All authors have contributed to read and approved the final version of the manuscript.

## Funding

This research project was financed with CHF 43,000 by the SAMS with means from the Käthe-Zingg-Schwichtenberg-Fonds (KZS Seed Grant 11/20) for the promotion of research in clinical ethics and public health ethics. The UPK Basel covers the costs associated with the infrastructure. The funding parties have neither a role in study design, data collection, and analysis nor in the decision to publish or preparation of the manuscript.

## Conflict of interest

The authors declare that the research was conducted in the absence of any commercial or financial relationships that could be construed as a potential conflict of interest.

## Publisher's note

All claims expressed in this article are solely those of the authors and do not necessarily represent those of their affiliated organizations, or those of the publisher, the editors and the reviewers. Any product that may be evaluated in this article, or claim that may be made by its manufacturer, is not guaranteed or endorsed by the publisher.

## Abbreviations

CRF: case report form
DMC: decision-making capacity
EKNZ: Ethics Committee Northwest and Central Switzerland
FMH: Swiss Medical Association
HRO: Ordinance on Human Research
PAD: physician-assisted dying
SACS: Staff Attitude to Coercion Scale
SAMS: Swiss Academy of Medical Sciences
StGB: Swiss Penal Code
UPK: University Psychiatric Clinics
USB: University Hospital Basel.
